# Notes and News

**Published:** 1895-09-07

**Authors:** 


### Notes and News.

(Collected and Communicated.)

The late Mr. John Alexander, of the firm of Messrs. William Baird and Co., has bequeathed £30,000 for the purpose of erecting a hospital at Coatbridge.

The foundation-stone of the new wing which is being added to the Devon and Exeter Hospital at a cost of between £13,000 or £14,000 was laid last week.

The authorities of the Smithsonian Institution have awarded the Hodgkins Prize of 10,000 dollars to Lord Rayleigh and Professor Ramsey for their discovery of argon as a constituent element of the atmosphere.

The outbreak of cholera is gaining ground in Hawaii. Eight persons, Chinese and natives, died of the disease last week. The American-Australian line steamer "Monawai," arriving off Honolulu, was prevented stopping there, the United States Consul notifying to the captain of the vessel that cholera had definitely broken out. Cholera is also still present in Japan and China.

The condition of the sick and wounded in the French army in Madagascar is giving rise to much anxiety. Certain regiments have suffered to an extraordinary degree. It is stated that engineering operations have proved very unhealthy, as is often the case in many malarious countries, disturbance of the soil having been provocative of disease. The hospitals are very full, they and the sanatorium containing about 5,000 men.

The new hospital for contagious diseases for Glasgow, now being constructed at Ruchill, will cover 38 acres, and will be surrounded by a wall nine feet in height. The wards are placed in 12 large buildings, each to accommodate 20 patients. Four small pavilions are provided for special cases. The hospital is intended to receive 408 patients. The institution is estimated to cost a quarter of a million. Lady Bell laid the foundation of the administrative block on Thursday.

An Exhibition of Drugs, Chemicals, and Medical Appliances, &c., organised by the *British and Colonial Druggist*, will be held at the Royal Agricultural Hall from September 10th to 13th.

A new children's ward was opened last week at the Bideford Hospital. A new board-room and further out-patients' accommodation has also been added, the whole at an expenditure of £767.

It is remarked in an American medical journal that London alone, of all the great cities of the world, has failed to establish a lead as an educational centre for medical students. The same journal gives the precedence to Berlin at the present time, whilst allowing Edinburgh to enter the lists as a rival.

The Session at the Medical School of the London Hospital will open on October 1st. In the afternoon a presentation will be made to Dr. Hughling Jackson, F.R.S., of his portrait in oils, by Lance Calkin, and a service of plate, on his retirement from the active staff of the hospital. The portrait will be hung in the college, and a replica will be given to Dr. Hughling Jackson. The prizes for 1894-95 will be distributed at the same time, and also the nurse-probationers' prizes. Sir James Paget will preside. The hospital dinner will take place on the same evening at the Criterion Restaurant.

The Middlesex Hospital has sustained a loss in the death of Mr. John Bell Sedgwick, who was deputy chairman and a member of the weekly board for thirty-seven years. Mr. Sedgwick has left £200 to the general funds. A sub-committee has been appointed to prepare plans for the new cancer wing for the above hospital. It is hoped that arrangements will shortly be made with the authorities of the medical school as to the joint use of the proposed site. The new building for the convalescent home in connection with the hospital is reported to be progressing favourably. A further sum of £3,333 has now been received from the trustees of Mr. Spicer's will. This amount has been lent on mortgage to meet the expenditure for this building.

The British Dental Association held its annual meeting in Edinburgh on Thursday. In his address from the presidential chair, Mr. W. Bowman Macleod advocated the establishment of a chair of clinical dental surgery. The claim of the profession for direct representation on the General Medical Council is still before the Privy Council. The report of the committee on the condition of the teeth of school children showed 11,422 as having been examined. This investigation, to which much interest has been attached, shows that the teeth of the children of the rich seem more liable to decay than those of children of the poorer classes. During the conference many demonstrations in practical dentistry were given and experiments made. On Saturday the value of anæsthetics was discussed at some length. Mr. Hewett, anæsthetist to several London hospitals, read a paper on "Chloroform in Dental Surgery." In this he made reference to the relative values of chloroform and ether, expressing his opinion in strong terms that the former should only be used in very exceptional cases. Mr. Hewett further stated that ether was an equally satisfactory agent, besides being considerably safer.

